# Comparison of food and beverage products’ availability, variety, price and quality in German and US supermarkets

**DOI:** 10.1017/S1368980020002645

**Published:** 2020-12

**Authors:** Nanette Stroebele-Benschop, Kerstin Wolf, Katharine Palmer, Casey J Kelley, Stephanie B Jilcott Pitts

**Affiliations:** 1 Department of Nutritional Psychology, Institute of Nutritional Medicine, University of Hohenheim, 70593 Stuttgart, Germany; 2 Department of Public Health, East Carolina University, Greenville, NC, USA

**Keywords:** Supermarket, Product availability, Price, Variety, Fruits, Vegetables

## Abstract

**Objective:**

To assess availability, variety, price and quality of different food products in a convenience sample of supermarkets in Germany and the USA.

**Design:**

Cross-sectional study using an adapted version of the Bridging the Gap Food Store Observation Form.

**Setting:**

Information on availability, quality, price and variety of selected food products in eight German and seven US supermarkets (discount and full service) was obtained and compared by country.

**Results:**

A general tendency for lower prices of fruits and vegetables in Germany was observed, while produce quality and variety did not seem to differ between countries, with the exception of the variety of some vegetables such as tomatoes. Chips and cereals did not differ significantly in variety nor price. In both countries, high energy-dense foods were lower in energy costs than lower energy-dense foods.

**Conclusions:**

The influence of food prices and availability on consumption should be further explored, including the impact of country differences.

One main determinant of today’s high prevalence of overweight and obesity is food intake, and a factor that greatly influences food choice and dietary patterns is the food environment^(1)^. Glanz *et al.*^(2)^ conceptualise both the community food environment (food venues located in the community) and the consumer food environment (the foods and beverages available in each food venue). Access and proximity to food stores, or the community food environment^(3–6)^, and the food store environment itself (consumer food environment) have been associated with the consumption of certain food groups such as fruits and vegetables^(7–11)^. Product variety available in supermarkets may be associated with intake^(12,13)^. In addition, pricing of food items is a determining factor in consumption patterns. High energy-dense foods (such as fats and sweets) are often less expensive per calorie than lower energy-dense foods including fresh fruits, vegetables and meats^(14–16)^. Taken together, the evidence suggests that access and availability to healthy and affordable foods in the consumer food environment influence consumers’ diet^(13,17,18)^.

National data indicate no differences in the intake of fruits and vegetables^(19,20)^ or even sugar and fat^(21,22)^ between Germans compared with Americans. Both Americans and Germans regularly consume above recommended amounts of meat and less than the recommended amount of vegetables^(23)^. However, there is a higher prevalence of overweight and obesity in the USA *v.* Germany^(24)^. These differences may be due to dietary factors including cultural eating patterns (e.g., US residents eat more meals away from home)^(25,26)^ and may also be due to physical activity-related factors^(27)^. Differences such as the percent of the budget spent for food away from home between these two countries likely play a key role^(28)^.

A study by Drewnowski *et al.*^(29)^ found that shopping at a lower-cost supermarket was associated with higher BMI when examining participants from studies in Seattle, Washington, USA and Paris, France. This suggests the importance of examining the food environment of discount supermarkets as well as larger, chain supermarkets, to determine potential reasons for these differences. Drewnowski *et al.*^(29)^ also emphasise the importance of studying the food environment across various cultures, in order to provide insights on the potential contributions of the consumer and community food environments with regard to unhealthy dietary patterns and obesity.

Availability and pricing of healthy foods have rarely been compared across countries as a possible explanatory factor for differences in consumption patterns and weight status^(30)^. Two recent comparisons of the nutritional content of beverages and children’s breakfast cereals available in supermarkets in New Zealand, Australia, Canada, USA and the UK revealed substantial differences in mean energy between countries^(31,32)^. Another recent study looked at relative energetic prices of food categories across different countries and concluded that relative food price variations across countries might explain international differences in the prevalence of undernutrition and overweight^(33)^. While pricing has been explored in a few studies, the authors are not aware of any study that examined possible differences in available food variety or quality within food categories between countries.

Therefore, the aim of the current study was to examine differences in availability, quality, price and variety of different food products in supermarkets in Germany and the USA, examining both full-service and discount supermarkets, to understand potential consumer food environment differences that might contribute to unhealthy eating patterns.

## Methods

### Study setting

Supermarket audits were conducted in a German city (634 830 inhabitants in 2019) and a city in North Carolina, USA (92 156 inhabitants) by two researchers (each) in eight supermarkets in Germany (four full service of two different retailers, four discount of two different retailers) and seven supermarkets in the USA (three full service of two different retailers, four discount of two different retailers). The location of the supermarkets can be described as medium to high social economic areas. Both supermarket types were chosen as store settings using the distinctions already used by other researchers^(34,35)^. The two selected discount retailers were the same for both countries, while the full-service retailers were not, due to the fact that there are not the same chain supermarkets in both countries. In both countries, full-service supermarkets are most often used by consumers to buy groceries while discount supermarkets are much more popular in Germany compared with the USA^(36)^. In general, grocery stores are smaller in size in Germany with less walking space, more consumer visits per week and less often located on the outskirts of the cities^(37,38)^.

In both countries, two researchers conducted their assessments between Tuesday and Thursday between 9:30 and 16:30 h during March through May of 2019 (discounter on one day, full service on another day). It took between 30 and 90 min to complete each audit depending on store size.

### Measures

For the assessment, an adapted version of the Bridging the Gap Food Store Observation Form^(39)^ was used. It was developed with input from several experts and adapted from validated tools such as the Nutrition Environment Measures Survey in Stores and the Communities of Excellence in Nutrition, Physical Activity and Obesity Prevention Food Availability and Marketing Survey^(40,41)^. The audit has been previously used in several studies assessing grocery stores and supermarkets^(42,43)^. It was selected because it was validated in the USA and includes foods and beverages that are considered both healthy (fruits and vegetables) and less healthy (sugary beverages). The audit includes assessment of availability and price of several food and beverage items, with an emphasis on fruits, vegetables and sugary beverages, which are important elements of the consumer food environment. The prices of all selected products as well as the cheapest available option (e.g., for cola) were recorded. Interior and exterior store characteristics are also included. The inter-rater reliability of the tool ranged from 0·84 for food and beverage product availability, and intra-class correlation coefficient (ICC) was 0·82 for produce pricing and 0·90 for counts of fresh, frozen and canned fruit and vegetable options^(39)^. For the study purpose, the section on items available at check-out, number of cash registers, exterior marketing, store exterior features (e.g., parking) or tobacco products were not taken into account for the store audit. Many of these aspects cannot be compared across these two countries (e.g., many grocery stores in German cities have underground parking or no parking). In addition, brand or type details of selected products (e.g., orange juice – by ‘Minute Maid’, apple – ‘Red Delicious’) were not assessed given the country differences in brands and produce types.

Based on a pre-screening in a German supermarket on product availability, the following types of foods in Table 1 were evaluated regarding quality, price and variety. The description for the quality assessment can be found in the original Bridging the Gap Food Store Observation Form^(39)^. The food types were selected based on their comparability across the two countries. At each store, an overall quality measurement was recorded for each product type (see online Supplementary Material).

**Table 1:** Product family, type and sub-categories for the measured food products

Product family	Product type	Sub-category (if applicable)	Quality
Fruits (including organic)	Apples		Good, acceptable, bad
	Pears		
	Bananas		
	Grapes		
	Citrus fruits	Oranges	
Vegetables (including organic)	Tomatoes		Good, acceptable, bad
	Cucumbers		
	Peppers	Excluding chillies	
	Salads	Iceberg lettuce	
	Carrots		
Potato chips	All kinds		
Cereals	Cornflakes	Kellogg’s and store brand	
Sugary beverages	Soft drinks	Cola	
	Juice drinks		

### Statistical analyses

To assess the inter-rater reliability of the audits, ICC were estimated for the US and German audits. All numerical variables including estimates of quality were used for this calculation. ICC and 95 % CI were computed using a single measure reliability two-way model ANOVA estimating absolute agreement between the raters.

To allow for comparison between US and German supermarkets, prices in US dollars per pound ($/lb) were converted into euros per kilogram (€/kg). The exchange rate on the 26 April 2019 (1 US dollar = 0·89578 euros) was used, as this was within the period in which the audits were conducted. Prices of lettuce and cucumber were provided per item, so these were converted into euros per kilogram using the weights of these items in their respective countries since both products are sold by piece and not by weight.

Summary statistics were used to compute availability, variety, quality and price of fruits, vegetables, cereals, potato chips and sugary beverages from the adapted version of the Bridging the Gap-Community Obesity Measures Project Food Store Observation Form^(29,39)^. Averages, sd and two-sample independent *t* tests were calculated. Energy density graphs were created using estimates of energy density per gram of each food item, and calculating the energy costs by identifying the price in Euros for 418·4 kJ (100 kcal) of each item, averaged across all supermarkets in the audit. Statistics were run using R version 3.6.1.

## Results

The two-way, absolute agreement, single rater ICC was assessed for both countries. For Germany, all variables had an ICC of 0·999, 95 % CI 0·999, 0·999. For the USA, the ICC was 0·84, 95 % CI 0·764, 0·887.

### Fruit and vegetable variety

In both countries, all selected products were available. The variety of apple types was larger than the variety of types of pears, bananas, grapes or oranges (Table 2). The variety of available fruits did not differ significantly between the two countries.

**Table 2:** Variety of types of fruits and vegetables available in Germany and the USA, mean number (sd). Welch’s two sample *t* tests

	USA (*n* 7)		Germany (*n* 8)		
	Mean	sd	Mean	sd	*P*
Variety					
All available fruits	21·86	2·04	26·00	6·57	0·13
Apples	12·43	3·74	12·75	3·37	0·86
Pears	3·00	1·41	3·75	0·71	0·24
Bananas	2·43	0·53	2·88	0·99	0·29
Grapes	3·29	1·25	4·00	1·31	0·30
Oranges	4·43	1·62	4·50	2·73	0·95
All available vegetables	25·00	2·65	30·25	4·30	0·01**
Tomatoes	8·71	2·87	13·50	5·15	0·04*
Peppers	8·14	4·34	9·50	2·98	0·50
Carrots	7·57	4·39	5·12	0·99	0·20
Cucumbers	3·29	0·95	4·12	1·73	0·26
Salads	35·14	24·07	14·38	9·56	0·07
Potato chips	57·00	45·30	58·62	37·75	0·94
Cereals	114·00	106·92	41·25	31·71	0·13
Sugary beverages*	180·07	177·97	226·75	189·12	0·63
Price (€ per kg or l)					
All below fruits (*n* 5)	2·50	1·01	1·71	0·49	0·28
Apples	2·29	0·40	1·20	0·56	0·00***
Pears	2·45	1·03	1·70	0·74	0·14
Bananas	0·80	0·13	1·21	0·35	0·01**
Grapes	4·10	1·40	3·40	0·69	0·26
Oranges	2·87	2·09	1·02	0·13	0·06
All below vegetables (*n* 5)	1·90	0·43	1·76	0·36	0·77
Tomatoes	2·07	0·72	1·96	0·23	0·72
Peppers	3·27	0·68	2·59	0·60	0·06
Carrots	1·32	0·27	1·08	0·43	0·20
Cucumbers†	1·57	0·27	1·82	0·35	0·15
Salad†	1·27	0·23	1·37	0·19	0·39
Potato chips	5·73	5·36	4·40	1·13	0·54
Store brand cornflakes	2·80	1·38	1·98	0·01	0·17
Cheapest cola	1·00	0·76	0·76	0·09	0·44

* Soft drinks, < 100 % fruit juice and 100 % fruit juice.

† Adjusted for average size.

Regarding assessed vegetable variety, results show that significantly more types of tomatoes were offered in the German supermarkets compared with the US supermarkets (*t*(11·2) = −2·26), while salad variety in the USA exceed offered varieties in Germany albeit not significantly (*t*(7·7) = 2·14). The remaining assessed vegetables such as cucumber, peppers and carrots were offered in similar varieties in both countries, but overall vegetable variety was significantly higher in Germany compared with the USA (*t*(11·8) = −2·88).

### Fruit and vegetable prices

Supermarkets’ pricing per kg of fruit had the highest range for grapes in Germany, with the lowest price at €2·38 and the highest at €4·38. In the USA, oranges had the highest price variations between supermarkets, with the lowest at €1·72 and the highest price per kg at €7·48. Prices for fruit differed between the countries with lower prices for apples in Germany but higher prices for bananas.

Prices for vegetables appeared to be generally cheaper in German supermarkets compared with the US supermarkets; this seems to be particularly the case for peppers (*t*(12·0) = 2·04), carrots (*t*(11·9) = 1·34), salads (*t*(8·2) = 5·07) and cucumbers (*t*(12·3) = 2·15). The prices for tomatoes seem to vary widely between US supermarkets.

### Differences in food products’ variety, prices and quality across supermarkets

When comparing the different types of supermarkets (discount *v*. full service), in both countries the mean number of different types of fruits and vegetables was higher in full-service supermarkets. Discount supermarkets offered 21·12 (sd 1·64) varieties of fruits and 25·25 (sd 2·96) varieties of vegetables, compared with 27·43 (sd 6·11) varieties of fruits and 30·71 (sd 4·11) varieties of vegetables offered in full-service supermarkets. The differences were significant for both fruits (*t*(6·8) = −2·65, *P* = 0·03) and vegetables (*t*(10·8) = −2·92, *P* = 0·01) as well as the number of different types of potato chips (*t*(6·5) = −8·02, *P* = 0·00), cereals (*t*(6·1) = −3·45, *P* = 0·01) and sugar-sweetened beverages (*t*(6·5) = −15·33, *P* = 0·00).

There were no significant differences for the aggregated mean prices of the assessed fruits or vegetables between discount and full-service supermarkets. Only tomatoes and cola were significantly more expensive in full-service supermarkets (*t*(8·5) = −3·32, *P* = 0·01 and *t*(8·4) = −2·43, *P* = 0·04, respectively), with a mean price of €1·70 per kg of tomatoes in discount supermarkets compared with €2·37 in full-service supermarkets, and €0·61 per litre of cola in discount supermarkets compared with a mean price of €1·19 in full-service supermarkets.

Product quality between the supermarkets did not differ; no product type was identified as being of overall ‘bad’ quality and all of the US supermarkets offered ‘good’ quality fruits and vegetables. ‘Acceptable’ quality pears, oranges, tomatoes, cucumbers and peppers were found in some German supermarkets. This was independent of the type of supermarket, as nine acceptable quality product types were found in discounter supermarkets and eight acceptable quality product types in full-service supermarkets.

### Cereal, potato chips and sugar beverage variety and prices

For cereals, the variety of cereals was higher in the USA than Germany, but this was not significant. Price differences for store brand cornflakes were not significant; however, there was a higher range of prices among the cereals in the USA (between €1·73 and €5·11 per 1 kg) compared with German supermarkets (€1·98 or €1·99 per 1 kg in all stores).

Mean number of potato chip varieties for the two countries were similar, with fifty-seven varieties available in the USA and almost fifty-nine in Germany. Potato chips in German supermarkets were similarly priced, with only one full-service supermarket in Germany offering slightly higher priced potato chips at €0·72 per 100 g, compared with €0·40 per 100 g in all other supermarkets. There was a wider range in price for chips in US supermarkets, where the cheapest price for chips was €0·28 per 100 g, and the most expensive was €1·70 per 100 g in a full-service supermarket.

Variety of sugary beverages varied greatly between discount and full-service supermarkets in both the USA (between a mean of 27 sugary beverages in the discount supermarkets and a mean of 382 in the full-service supermarkets) and Germany (between 50 and 461 types available). The lowest number was in a US discount supermarket, with nineteen different types available and the highest number was in a US full-service supermarket, with 500 different varieties available. Prices for cola also varied considerably within US supermarkets, from €0·27 per the cheapest litre size bottle to €1·96 per bottle, while in Germany, prices across the tested supermarkets were relatively stable (€0·60 to €0·90).

**Fig. 1:**
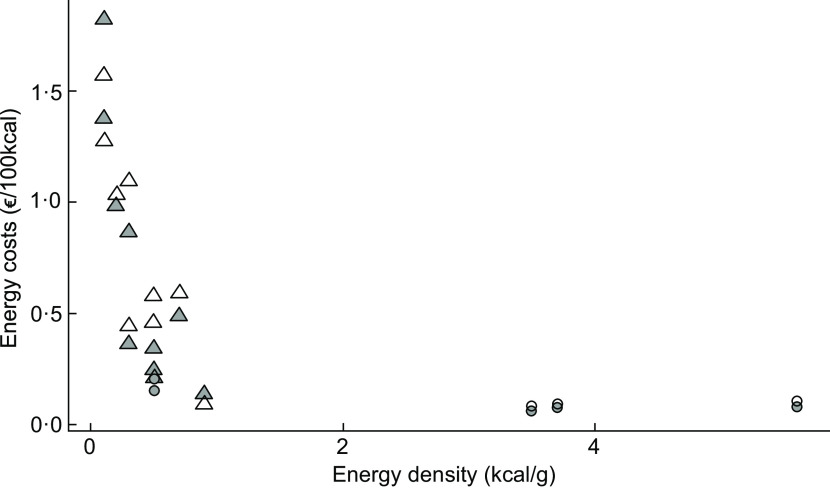
Proportion of energy density (kcal/g) to energy costs (€/100 kcal) across the assessed food products categorised into ‘healthy’ (less energy-dense foods such as fruits and vegetables) and ‘unhealthy’ (high energy-dense foods such as sweets or savoury snacks) using the mean prices across all supermarkets. (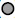), Germany; (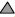), USA; (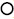), Unhealthy; (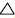) Healthy

When looking at energy density of the assessed food products, it was confirmed that fruits and vegetables in both countries have higher energy costs (€/100 kcal) than chips or cereals. The results were similar for both countries, see Fig. 1.

## Discussion

The current study assessed various food products’ variety, prices and quality in supermarkets in the USA and Germany using a supermarket audit tool which demonstrated strong inter-rater reliability for both countries. There was a tendency for lower prices of fruits and vegetables in Germany *v.* the US stores, while produce quality and variety did not differ, with the exception of the variety of some vegetables. Interestingly, the range in prices in German supermarkets was lower, which would explain findings that showed that, compared with the US and other countries^(44–46)^, in Germany, no differences in the food environment between high and low socioeconomic areas were found^(14)^. Furthermore, food product prices do not appear to be automatically cheaper in discount supermarkets in either country, questioning consumers’ assumption of a better deal in discount supermarkets. There is some research pointing in a similar direction mainly for selected everyday food items (e.g., milk, cola soft drink)^(47)^. But academic research in this area across all countries seems to be lacking.

It can be speculated that lower produce prices encourage greater produce intake. However, national data do not seem to show country differences in consumption of the studied food products^(19–22)^ whereas differences in the prevalence of overweight and obesity between the countries exist.

While the intake of fruits and vegetables appears similar in both countries, our study found that all food and beverages were higher cost, on average, in US *v.* German markets. This could be due to the differences in study setting as the US stores were located in a city with a smaller population and potentially fewer stores and thus less market competition, which may contribute to the higher prices for food in the US *v.* German stores that were assessed.

Overall, the calculation of energy costs between the assessed food products demonstrates that healthy products (less energy-dense foods such as fruits and vegetables) are more expensive than energy-dense products (e.g., chips) in both countries. However, in Germany, there were lower overall costs for fruits and vegetables, raising the question of the influence of energy costs as a key part of the consumer food environment that could affect population-based purchasing behaviour^(48)^. Additional studies are needed to understand the impact of food and beverage price on food consumption, particularly since price reductions and increased variety of healthy foods appear to increase consumption^(1,49)^. There are several studies demonstrating that price discounts on healthier items can promote healthier purchases^(50)^, and additional studies are needed across countries to determine the most effective in-store strategies to promote healthier purchases.

Limitations of the study include the small convenience sample of stores included, and the differences in the study settings. Possible seasonal effects and the lack of direct consumer data are additional weaknesses. Furthermore, the assessment of produce quality was purely subjective and is thus a limitation in the current study. Nevertheless, this is the first study using a tool adapted from a validated instrument in both chain and discount supermarkets in two distinct countries. It is also the first study to compare prices, energy density and energy costs of selected products across these two countries.

In conclusion, it is thought that availability, variety, price and quality of food products influence dietary intake. Reciprocally, when consumers demand higher quality, or healthier foods, these foods may be more available in stores. The reciprocal nature of supply and demand within food stores is complex but given their economic and political power in the food system, supermarkets could be impactful and positive levers to improve dietary quality in both countries^(51,52)^. Our study can be a model for future studies using larger and more representative samples of stores for assessment, with a goal of additional health promotion efforts in supermarkets.

## Supplementary material

For supplementary material accompanying this paper visit https://doi.org/10.1017/S1368980020002645. click here to view supplementary material
