# Supplementary material for: Comparison of food and beverage products’ availability, variety, price and quality in German and US supermarkets
Source: Public Health Nutr. 2020 Sep 11;23(18):3387–93. doi: 10.1017/S1368980020002645 (PMC7737043; doi:10.1017/S1368980020002645)
Supplement: Supplementary file 1 [file S1368980020002645sup001.pdf]

March/April 2019 (adapted BTG-FSOF)

Location: several locations possible<br>Price: cheapest option available, no sale<br>Q = Quality, a=good, b=OK, c=bad (1=yes, 0=no)<br>No. potato chips, cereals, SSBs: all varieties, pack sizes, flavors counted<br>Availability: yes=1, no=0	**grey fields to fill in**

	**Supermarket**				**Name**	**Name**
	**City (c)/Suburb (s)**					
	**Location**					
	**Date**					
	**weekday**					
	**Time**					
	**Walking direction**					
	**Information**					
**No**	**Element**	**sub-points**	**questions**	**Quality**		
	**Fruits**	Location in Supermarket				
**1**	**Fruits**	Variety of fruits				
**1**	**Fruits**	Number of organic options				
**1**	**Fruits**	% organic				
**1**	**Fruits**					
**1**	**Fruits**	Apples	Number of types			
**1**	**Fruits**		Price/lbs ($)			
**1**	**Fruits**		Quality	a		
**1**	**Fruits**			b		
**1**	**Fruits**			c		
**1**	**Fruits**					
**1**	**Fruits**	Pears	Number of types			
**1**	**Fruits**		Price/lbs ($)			
**1**	**Fruits**		Quality	a		
**1**	**Fruits**			b		
**1**	**Fruits**			c		
**1**	**Fruits**					
**1**	**Fruits**	Bananas	Number of types			
**1**	**Fruits**		Price/lbs ($)			
**1**	**Fruits**		Quality	a		

1	**Fruits**			b		
1	**Fruits**			c		
1	**Fruits**					
1	**Fruits**	Grapes	Number of types			
1	**Fruits**		Price/lbs ($)			
1	**Fruits**		Quality	a		
1	**Fruits**			b		
1	**Fruits**			c		
1	**Fruits**					
1	**Fruits**	Citrus Fruits	Number of varieties			
1	**Fruits**		Numer of ORANGE types			
1	**Fruits**		Price/lbs [$] (orange)			
1	**Fruits**		Quality	a		
1	**Fruits**			b		
1	**Fruits**			c		
1	**Fruits**					
1	**Fruits**	Comments				
2	**Vegetables**	Location in Supermarket				
2	**Vegetables**	Variety of vegetables				
2	**Vegetables**	Number of organic options				
2,0	**Vegetables**	% organic				
2	**Vegetables**					
2	**Vegetables**	Tomatoes	Number of types			
2	**Vegetables**		Price/lbs [$]			
2	**Vegetables**		Quality	a		
2	**Vegetables**			b		
2	**Vegetables**			c		
2	**Vegetables**					
2	**Vegetables**	Cucumber	Number of types			
2	**Vegetables**		Price/each [$]			
2	**Vegetables**		Quality	a		
2	**Vegetables**			b		
2	**Vegetables**			c		
2	**Vegetables**					
2	**Vegetables**	Peppers (w/o chili)	Number of types			
2	**Vegetables**		Price/lbs [$]			
2	**Vegetables**		Quality	a		

**2**	**Vegetables**			b		
**2**	**Vegetables**			c		
**2**	**Vegetables**					
**2**	**Vegetables**	Salad	Number of types			
**2**	**Vegetables**		Number of ready to eat			
**2**	**Vegetables**		Price/head iceberg [$]			
**2**	**Vegetables**		Quality	a		
**2**	**Vegetables**			b		
**2**	**Vegetables**			c		
**2**	**Vegetables**					
**2**	**Vegetables**	Carrots	Number of types			
**2**	**Vegetables**		Price/lbs [$]			
**2**	**Vegetables**		Quality	a		
**2**	**Vegetables**			b		
**2**	**Vegetables**			c		
**2**	**Vegetables**					
**2**	**Vegetables**	Comments				
**3**	**Potato Chips**	Location in Supermarket				
**3**	**Potato Chips**	Numer of potato chips				
**3**	**Potato Chips**	Price/5oz [$] (home brand)				
**3**	**Potato Chips**	Comments				
**4**	**Cereals**	Location in Supermarket				
**4**	**Cereals**	Number of cereals				
**4**	**Cereals**	Price/lbs [$] (Corn Flakes - Kellogs)				
**4**	**Cereals**	Price/lbs [$] (Frosties - Kellogs)				
**4**	**Cereals**	Price/lbs [$] (Corn Flakes - home brand)				
**4**	**Cereals**	Price/lbs [$] (Frosties - home brand)				
**4**	**Cereals**	Comments				
**5**	**SSBs**	Location in Supermarket				
**5**	**SSBs**	Numer of different SSBs				
**5**	**SSBs**	Numer of different SS-fruit nectars				
**5**	**SSBs**	Numer of different SS-energy drinks				
**5**	**SSBs**	Size of greatest bottle [l]				
**5**	**SSBs**	Price/l [$] (Cola, cheapest)				
**5**	**SSBs**	Comments				
